# Recurrent acromegaly: a systematic review on therapeutic approaches

**DOI:** 10.1186/s12902-023-01533-w

**Published:** 2024-01-26

**Authors:** Seyed Farzad Maroufi, Mohammadmahdi Sabahi, Seyed Sahab Aarabi, Mohammad Samadian, Rocco Dabecco, Badih Adada, Karla M. Arce, Hamid Borghei-Razavi

**Affiliations:** 1 Neurosurgery Research Network (NRN), Universal Scientific Education and Research Network (USERN), Tehran, Iran; 2 Faculty of Medicine, Tehran University of Medical Sciences, Tehran, Iran; 3 Department of Neurological Surgery, Pauline Braathen Neurological Centre, Cleveland Clinic Florida, Weston, Florida USA; 4 Department of Neurosurgery, Loghman Hakim Hospital, Shahid Beheshti University of Medical Sciences, Tehran, Iran; 5 Department of Endocrinology Diabetes and Metabolism, Cleveland Clinic Florida, Weston, Florida USA; 6 Department of Neurological Surgery, Pauline Braathen Neurological Centre, 2950 Cleveland Clinic Blvd., Weston, FL 33331 USA

**Keywords:** Acromegaly, Radiotherapy, Medical therapy, Trans-sphenoidal surgery, Recurrence

## Abstract

**Background and objective:**

Management of recurrent acromegaly is challenging for both neurosurgeons and endocrinologists. Several treatment options including repeat surgery, medical therapy, and radiation are offered for such patients. The efficacy of these modalities for the treatment of recurrence has not been studied previously in the literature. In this study, we aim to systematically review the existing cases of recurrence and come to a conclusion regarding the appropriate treatment in such cases.

**Method:**

A systematic review was performed through PubMed, Scopus, Web of Science, and Cochrane database to identify studies reporting the treatment outcome of recurrent acromegaly patients. Using PRISMA (Preferred Reporting Items for Systematic Reviews and Meta-Analyses) guidelines, the included studies were reviewed for primary and secondary treatment, complications, and outcomes of the secondary treatment.

**Results:**

The systematic review retrieved 23 records with 95 cases of recurrent acromegaly. The mean time of recurrence was 4.16 years after the initial treatment. The most common primary treatment was surgery followed by radiotherapy. The remission rate was significantly higher in medical and radiotherapy compared to surgical treatment.

**Conclusion:**

In cases of recurrent acromegaly, the patient may benefit more from radiotherapy and medical therapy compared to surgery. As the quality of evidence is low on this matter feature studies specifically designed for recurrent patients are needed.

## Background

Acromegaly is a rare disease, with a prevalence ranging from 2.8 to 13.7 cases per 100,000 population [1]. It is a slowly progressive disease characterized by overproduction of growth hormone (GH) and insulin-like growth factor 1 (IGF-1), typically due to GH-secreting adenoma [2]. The nature of the acromegaly often leads to a diagnostic delay of 5-10 years after symptoms onset. Morbidities associated with acromegaly, such as sleep apnea, cardiomyopathy, and glucose intolerance—contributing to a shortened life expectancy—tend to show improvement upon normalization of GH and IGF-1 levels [3]. Meanwhile, clinical presentations such as acral hypertrophy, coarse features, and arthropathy often persist after GH normalization [4].

Transsphenoidal pituitary surgery has been considered first-line therapy for acromegalic patients due to the ability to alleviate mass effects while inducing an immediate “remission” secondary to tumor removal. While studies have reported a good remission rate for transsphenoidal surgery, 2-3% of patients may experience recurrence. Recurrence is defined as the return of GH hypersecretion after an initial satisfactory course of therapy. Although the recurrence of phenotypic signs and symptoms may indicate the return of acromegaly, a biochemical diagnosis is required [5]. While the incidence of acromegaly recurrence is presumed to be low, earlier studies overestimated the recurrence incidence. This overestimation occurred because previous remission criteria misclassified patients with persisting postoperative disease [6]. Therefore, it is crucial to differentiate between true recurrences and persistent disease resulting from unsuccessful surgery when evaluating surgical success in acromegaly [7]. Furthermore, various predictors of recurrence in acromegalic patients have been identified, such as young age, larger tumors, aggressiveness, and elevated pre- and postoperative hormone levels [8–10].

For patients with recurrent acromegaly, several therapeutic options are available, including repeat surgery, radiotherapy, and medical therapy. In case of recurrence, reoperation is reserved for patients with a visible lesion on MRI that is surgically accessible [11]. In patients without visible lesions on MRI or with inaccessible lesions, medical and radiotherapeutic approaches are the main treatment options. Among various radiotherapeutic approaches, conventional radiotherapy (CRT), [12] stereotactic radiotherapy (SRT), [13] and stereotactic radiosurgery (SRS) [14] are commonly used in acromegalic patients. Similarly, several medications are commonly used when considering medical therapy in acromegaly, including somatostatin analogs, [15] dopamine agonists alone, [16] or in combination with somatostatin analogs [17], and GH receptor antagonists [18]. This systematic review aimed to determine the most appropriate therapeutic modality for recurrent acromegalic patients. 

## Material and methods

### Literature search

This systematic review was conducted according to the Preferred Reporting Items for Systematic Reviews and Meta-Analyses (PRISMA) guideline. A systematic search of online databases (PubMed, Scopus, Web of Science, and Cochrane) was performed by March 2022, using the following terms and their equivalents: Acromegaly AND Adenoma AND Recurrence. 

### Study selection

The inclusion criteria were as follows: 1) studies including patients with recurrent acromegaly after initial treatment, and 2) studies reporting previous and current treatment modalities and outcomes of these patients. Exclusion criteria were: 1) non-original articles and case reports, 2) lack of clinical data regarding treatment modalities and outcomes, and 3) studies not reporting outcomes for each modality. 

Two reviewers (S.F.M and S.S.A) independently assessed the titles, abstracts, and full texts of the identified records based on the stipulated eligibility criteria. Any conflicts were resolved by another author (M.M.S) before proceeding to the next step. Additionally, the reference lists of the included studies were manually searched for any additional relevant records. 

### Data extraction

Data extraction was performed using a standardized Excel datasheet by one of the reviewers (S.F.M). Two other reviewers, independently, rechecked the extracted data (S.S.A and M.M.S). The outcomes of interest were remission rate, complications, and mortality associated with each treatment modality for recurrent acromegaly. 

### Definitions

Recurrence was defined by the reappearance of biochemical, clinical, or imaging findings consistent with the recurrence of acromegaly. Remission was defined as the normalization of IGF1 and GH levels, adhering to the criteria specified in each study. Specific definitions of recurrence and remission, as provided in each article, are outlined in Table 1.

**Table 1:** Summary of the included studies for systematic review

**Study characteristics**					**Initial treatment**		**Secondary treatment detail**		
**Author**	**Year**	**Patients**	**Criteria for remission**	**Criteria for recurrence**	**First therapeutic modality**	**Time to recurrence (years)**	**Candidates**	**Modality**	**Remission**
Ross [19] 1988		5	Random GH<5ng/ml	Biochemical and/or clinical signs of active acromegaly after a period of time with normal GH levels	Surgery	NR	1	Surgery	1
							2	Radiotherapy	2
							1	Medical	1
							1	Expired	
Salinger [20] 1992		3	Relief of presenting symptoms and favorable changes in radiologic and serum endocrine studies	Second presentation of symptoms or regrowth of tumor after initial response	Surgery + Radiotherapy	NR	1	Surgery	1
							2	Surgery + Radiotherapy	2
Long [21] 1996		1	Random GH<5µg/L and OGTT<2µg/L	NR	Surgery	4.83	1	Surgery	1
Abosch [22] 1998		9	GH<5ng/mL within the first 30 days following surgery	An initial remission followed by a rise in GH levels + recurrent symptoms	Surgery	3.3	3	Radiotherapy	2
							2	Medical	2
							4	No treatment	
Freda [23] 1998		10	A normal IGF-I level or OGTT<2ng/mL	An elevation of IGF-I or OGTT>2ng/ml subsequent to postoperative documentation of normalization	Surgery	NR	10	Surgery	4
Swearingen [24] 1998		5	Random GH<2.5ng/mL, OGTT<2ng/mL, Serum IGF-I normalization	Biochemical recurrence	Surgery	5	3	Surgery	1
							1	Medical	1
							1	Expired	
Biermasz [25] 2000		5	Random GH<2.5ng/mL, OGTT<1ng/mL, Serum IGF-I normalization	Biochemical recurrence	Surgery	6	2	Radiotherapy	2
							3	Medical	3
Pollock [26] 2002		1	Random GH<2ng/mL, Serum IGF-I normalization	Abnormal IGF-1 after normalization	Radiotherapy	1	1	Medical	1
Acosta-Gomez [27] 2005		3	Normalization of clinical sings (including basal and dynamic responses of GH) at 1 year after therapy with no evidence of residual tumor on CT or MRI	Clinical, biochemical and/or neuroradiological signs of tumor activity detected after therapy	Surgery	NR	1	Radiotherapy	1
							1	Medical	1
							1	No treatment	
Landolt [28] 2006		1	GH and IGF-I normalization	NR	Surgery + Radiotherapy	20	1	Radiotherapy	1
Petit [29] 2007		1	Serum IGF-I normalization	Abnormal IGF-I after remission	Surgery	12	1	Radiotherapy	0
Jagannathan [30] 2008		3	Serum IGF-I normalization	NR	Radiotherapy	NR	1	Radiotherapy	1
							2	Medical	2
Losa [31] 2008		1	Serum IGF-I normalization, Basal GH<2.5µg/L	Basal GH>2.5µg/L, Abnormal IGF-1	Surgery	NR	1	Medical	1
Yamada [32] 2010		2	Basal GH<2.5µg/L, OGTT<1ng/mL, Serum IGF-I normalization	NR	Surgery	NR	2	Medical	2
Albarel [33] 2013		10	Serum IGF-I normalization, OGTT<0.4µg/L 3 months post-surgery	Elevated IGF-1 levels or OGTT>0.4µg/L during follow-up.	Surgery	More than 0.5	2	Radiotherapy	2
							5	Medical	5
							3	No treatment	1
Van Rompaey [34] 2013		6	Serum IGF-I normalization, OGTT<1 ng/ml	NR	Surgery	NR	1	Surgery	1
							1	Radiotherapy	1
							4	Medical	4
Sankhla [35] 2013		1	NR	NR	Surgery + Radiotherapy	NR	1	Surgery	1
Shirvani [36] 2014		14	Basal GH<2.5µg/L, Serum IGF-I normalization	NR	Surgery	2.62	6	Surgery	4
							5	Radiotherapy	5
							3	Medical	3
Sun [37] 2014		4	Random GH<1μg/L, OGTT<0.4μg/L	NR	Surgery	NR	4	Surgery	1
Sinha [38] 2014		2	basal serum Growth Hormone<1 g/L	Hypersecretion after normalization	Surgery + Radiotherapy	NR	2	Surgery	2
Paluzzi [39] 2014		2	Serum IGF-I normalization	NR	Surgery	1.25	2	Surgery	2
Losa [40] 2017		4	NR	NR	Radiotherapy	NR	1	Surgery	1
							2	Radiotherapy + Medical	2
							1	Medical	1
Ismail [41] 2020		2	Serum hormone level normalization	NR	Surgery	NR	2	Surgery	2

*NR* Not reported, *OGTT* GH after oral glucose tolerance tests, *GH* Growth-hormone, *IGF-I* Insulin-like growth factor 1

### Risk of bias assessment

A modified version of the Joanna Briggs Institute (JBI) Critical Appraisal tool was used for quality appraisal. Three questions of the original questionnaire were deemed not applicable, as only a small proportion of patients in each study were included in our analysis. These questions were, “Did the case series have consecutive inclusion of participants?”, “Did the case series have complete inclusion of participants?”, and “Was statistical analysis appropriate?”. 

### Statistical analysis

All analyses were conducted in R statistical analysis software (version 4.1.2, R Foundation for Statistical Computing) using the “meta” package. Remission rates were calculated using the “metarprop” function and generic inverse variance. Mean and range were used to report the quantitative data. The chi-square test was used to assess the difference in remission in each modality due to the low quality of evidence and limited number of patients. A P-value less than 0.05 was considered significant. 

## Result

**Fig. 1:**
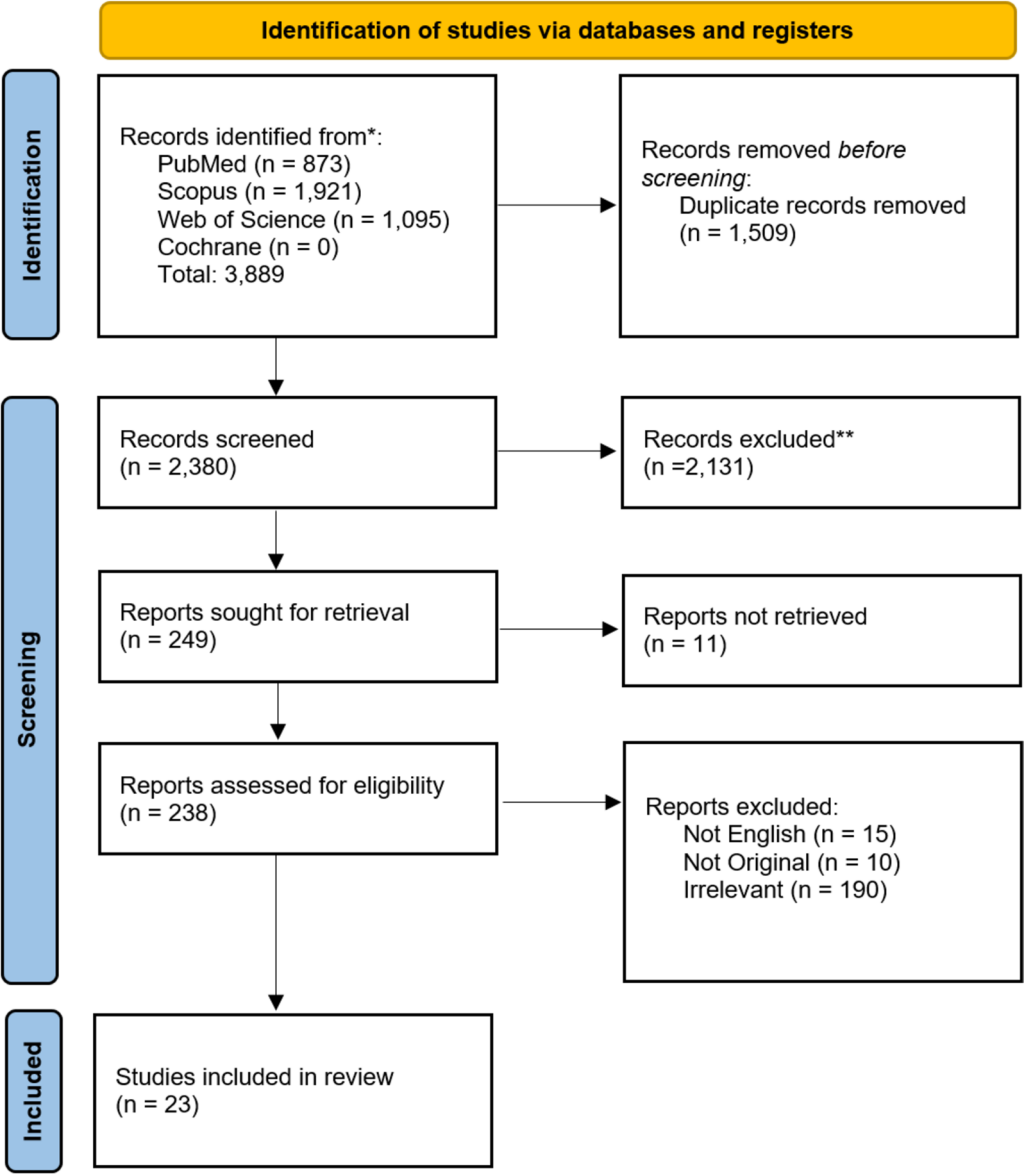
The PRISMA flow diagram

Our search strategy yielded a total of 3,889 records. After removing duplicates, 2,380 records underwent title and abstract screening. After title and abstract screening, 249 records were assessed for eligibility by reviewing their full texts. Lastly, 23 articles were included for quantitative analysis. The PRISMA flow diagram is depicted in Fig. 1.

All the included studies were case series, except for one case-control study. The publication dates ranged from 1988 to 2020, collectively covering 95 patients identified as recurrent cases of acromegaly. The criteria for acromegaly diagnosis were GH level in 6 studies, GH and IGF-1 level in 13 studies, and IGF-1 level in 2 studies, and the criterion was not available in 2 studies. The criterion for recurrence was biochemical recurrence in the majority of studies, although the exact criterion was not reported in some of the included studies. Demographic information and postoperative complications were often unavailable and were consequently omitted from the analysis. 

The mean time of recurrence was 4.16 years after the initial treatment. Surgery was the most frequently performed initial procedure (80 patients), followed by radiotherapy (8 patients) and a combination of radiotherapy and surgery (7 patients). Detailed information regarding treatments in each study is demonstrated in Table 2.

**Table 2:** Quality assessment ratings for the included studies

Author	Ross	Salinger	Long	Abosch	Freda	Swearingen	Biermasz	Pollock	Acosta-Gomez	Landolt	Petit	Jagannathan	Losa	Yamada	Albarel	Van Rompaey	Sankhla	Shirvani	Sun	Sinha	Paluzzi	Losa	Ismail
Were there clear criteria for inclusion in the case series?	Y	N	Y	Y	U	Y	Y	Y	N	Y	N	Y	Y	N	Y	Y	N	U	Y	Y	N	Y	Y
Was the condition measured in a standard, reliable way for all participants included in the case series?	Y	U	Y	Y	Y	Y	Y	Y	U	U	U	Y	Y	Y	Y	Y	N	Y	U	Y	Y	N	N
Were valid methods used for identification of the condition for all participants included in the case series?	U	U	Y	U	U	U	Y	Y	U	U	Y	Y	U	Y	Y	N	N	N	N	Y	N	Y	U
Was there clear reporting of the demographics of the participants in the study?	N	N	Y	N	N	N	N	N	N	Y	N	N	N	N	N	N	N	N	N	N	N	N	N
Was there clear reporting of clinical information of the participants?	Y	Y	Y	Y	Y	U	Y	Y	Y	Y	Y	Y	Y	Y	U	U	Y	U	Y	Y	Y	Y	U
Were the outcomes or follow up results of cases clearly reported?	Y	Y	Y	Y	Y	Y	Y	Y	Y	Y	Y	Y	Y	Y	Y	Y	Y	Y	Y	Y	Y	Y	Y
Was there clear reporting of the presenting site(s)/clinic(s) demographic information?	N	N	N	N	N	N	N	N	N	N	N	N	N	N	N	N	N	N	N	N	N	N	N

*N* No, *Y* Yes, *U* Unclear

**Fig. 2:**
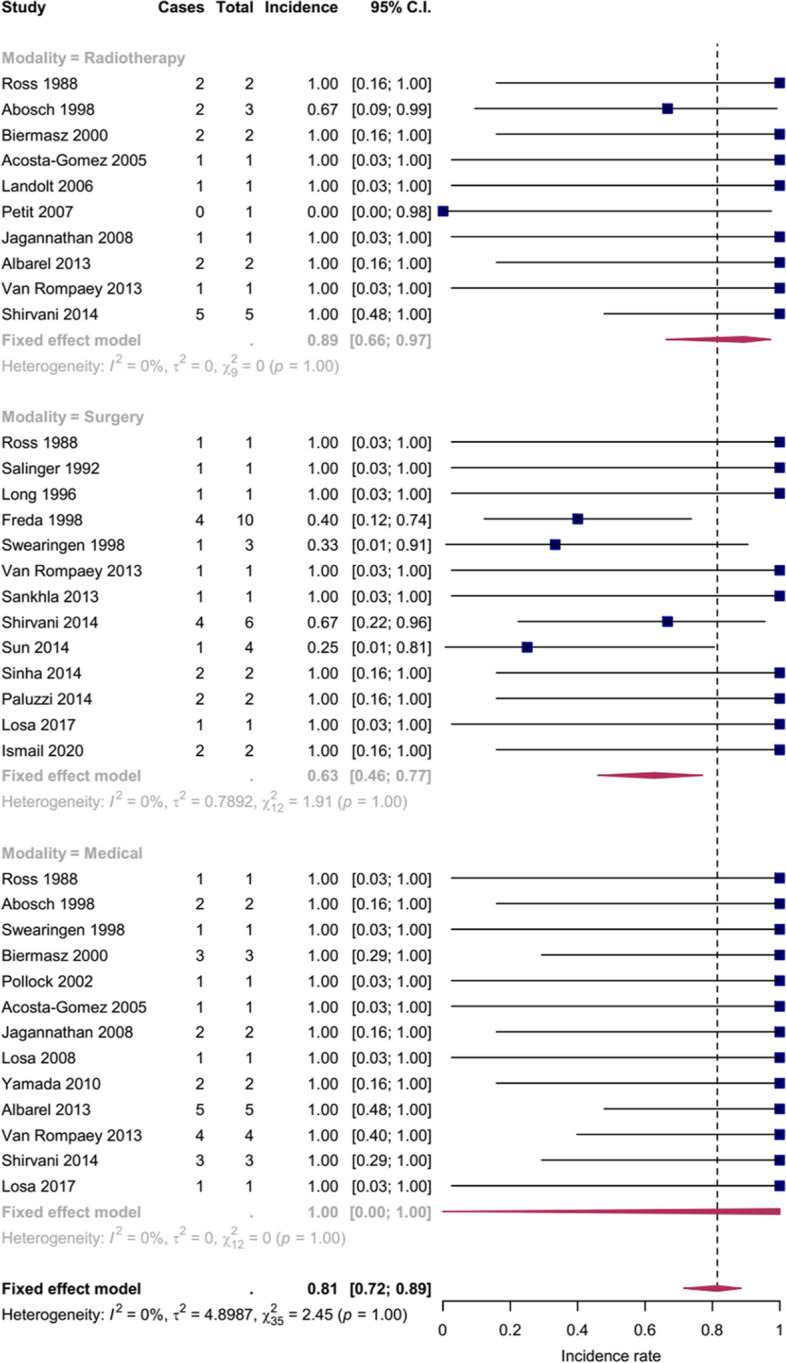
Forest plot of the remission rates pooled analysis

Secondary treatments were categorized into surgical treatment (35 patients), medical treatment (27 patients), and radiotherapy (19 patients). Only four patients underwent combination therapy (radiotherapy + surgery: 2 and radiotherapy + medical: 2) and were omitted from the analysis. Medical treatment had the highest remission rate, followed by radiotherapy and surgery, respectively. The remission rate was significantly higher in medical and radiotherapy modalities compared to surgical treatment, *P*<0.01 and *P*=0.03, respectively. The remission rates and confidence intervals for each modality are plotted in Fig. 2.

Considering the publication timeline, studies were divided into subgroups based on their year of publication (before 2000, 2000-2010, and after 2010). While no significant differences were observed in the first two subgroups (*P*=0.12, 0.18, respectively), a significant difference in remission rates emerged for studies published after 2010 (*P*=0.04). Notably, the 2000-2010 subgroup lacked reoperation cases, allowing a comparison only between medical and radiotherapeutic approaches. 

Quality assessment revealed that most studies had low-quality reporting of data and outcomes. Detailed ratings for each study are provided in Table 2. 

## Discussion

Recurrence of acromegaly following an effective surgical resection is uncommon (2–3%), but persistence of GH hypersecretion is frequently observed (43%) [3]. Following initial treatment failure or recurrence, acromegaly patients may undergo medical therapies, repeat surgery, or radiotherapy. This systematic review aims to compare the effectiveness of each of these modalities in patients with recurrent acromegaly. Although each treatment has its advantages and disadvantages, our analysis demonstrated that medical therapy and radiotherapy are superior to repeat surgery in terms of remission rate. 

The studies included in our review predominantly utilized both IGF-1 and GH levels for diagnosing and assessing remission in acromegaly. While earlier studies focused more on GH levels, IGF-1 measurement is a crucial part of the contemporary biochemical evaluation of acromegaly. IGF-1 is an excellent disease marker and its normalization corresponds with the improvement of other metabolic and mortality-related indicators in acromegaly [42–45]. Moreover, it should be noted that the concept of "biochemical remission" has undergone several modifications, with the GH threshold level gradually declining over time [3]. Hence, it is important to simultaneously monitor GH and IGF-1 levels in follow-up visits [45–47]. Freda et al., in a short-term follow-up of surgically treated acromegaly patients with discordant IGF-1 and GH levels, revealed that recurrence was more prevalent in those with abnormal GH suppression than those with normal GH [6]. Although none of the subsequent investigations found a higher recurrence rate in this group [48–50], a long-term prospective study by the same author reaffirmed the earlier result regarding the association between GH suppression and acromegaly recurrence [6, 51]. Moreover, recurrence may develop over a significantly longer period as recurrences have been recorded to occur up to 12 years following surgery [52]. According to the current criteria, surgical series indicate low recurrence rates of 0–2.4% [53, 54], but possibly extended follow-up is needed to detect all recurrences. 

Most studies in our analysis utilized gamma knife radiosurgery (GKRS) as a preferred radiotherapeutic approach for recurrent acromegaly. Landolt et al. compared single-fraction GKRS with conventional fractionated radiotherapy for recurrent acromegaly after surgical treatment and found GH normalization to occur more quickly in the GKRS group [14]. Recently, the use of conventional radiotherapy to manage recurrent acromegaly has been limited due to hypopituitarism, visual neuropathy, and secondary cerebral malignancy [3]. Meanwhile, stereotactic procedures (SRS and SRT) reduce the risk of subsequent malignancy and are more potent than conventional radiotherapy, but still result in similar rates of hypopituitarism [3, 55]. Pituitary insufficiency with an incidence of 0%-19% following GKRS, remains the most common adverse event of all radiotherapeutic approaches, typically manifesting within a year of treatment [14, 26, 56]. Further, patients with cavernous sinus invasion appear to be more susceptible to delayed hypopituitarism [57]. Lastly, the anti-secretory impact of SRS on GH production is delayed less (2–3 years) than that of SRT (5–10 years), but SRS cannot be employed if the tumor is within 3 mm of the optic chiasm [58]. 

Reoperations in persistent or recurrent acromegaly may be associated with lesser remission and greater complication rates [21]. Consistently, we found reoperation to have a significantly lower remission (63%) compared to other modalities. This remission rate is higher than the remission rate yielded for reoperation of the combined recurrent and persistent GH-secreting adenomas (46.8%) in a systematic review by Almeida et al. [59]. This difference might be due to the inclusion of only recurrent cases in our study. Moreover, the likelihood of experiencing a complication may rise in case of reoperation due to scars of previous operations [60]. Hence, the decision to perform repeated surgery should be individualized, recommending repeated surgery when there are no alternative options or a significant tumor burden in a relatively "safe" resection area [61]. Yamada et al. suggested reoperation in acromegalic patients with persistent or recurrent disease without adequate response to adjuvant therapy, intolerance to the treatment, or financial concerns [32]. The latter study also noted that noninvasive adenomas posing relatively low complication risks are more likely to be treated surgically than those that invade the cavernous sinus, especially in young individuals [32]. Nevertheless, it is encouraged to debulk and lower GH levels to increase the likelihood of remission with postoperative adjuvant therapy and ameliorate mass effect symptoms [32].

GH-suppressing medications are potent therapeutic options. Among many medications, somatostatin analogs have proven to be more efficient in treating persistent or recurrent acromegaly than dopamine agonists [17]. Meanwhile, dopamine agonists are more effective at treating acromegaly in prolactin co-secretion [17]. In cases when other medical treatments or radiotherapy have failed, GH receptor antagonists can normalize IGF-1 levels in up to 95% of patients within the first year [62]. Nevertheless, prolonged consumption of these medications, due to their suppressive and non-curative nature, may lead to economic and health-related concerns [63]. Each medical therapeutic option is associated with a specific group of adverse events, dopamine agonists with dizziness, gastrointestinal discomfort, and hypotension; GH receptor agonists with gastrointestinal discomfort, skin reactions, and abnormal liver enzymes; and somatostatin analogs with abdominal pain, diarrhea, and cholelithiasis [64, 65]. Recent studies have focused on combination therapy to decrease the adverse events associated with each medical treatment and increase their efficacy [63]. Considering our results demonstrating the superiority of medical therapy over other options, we believe future studies should focus on cost-effectiveness and comparing different medications. Moreover, considering the effectiveness, costs, and adverse events associated with each medication, a tailored approach should be utilized when approaching patients with recurrent acromegaly. 

Our study had several limitations. The scarcity of acromegaly recurrence leads to a small population found in the literature and hence weakens the power of analysis. Definitions of acromegaly, remission, and recurrence have changed over time, and different studies have used different criteria. The unavailability of demographic data regarding patients with recurrence limits the generalizability of our results to other populations. Residual tumor volume is the main indication for reoperation, but no study reported residual volume in recurrent cases. Hence, we believe the true remission rate may even be lower for reoperation. While a combination of treatment modalities is considered a suitable treatment option for recurrent acromegaly, we could not investigate combination therapy due to limited reported patients. Moreover, since most studies did not report complications after secondary surgery, we couldn’t compare the mentioned modalities from aspects other than remission rate. This study is the first of its kind, highlighting the probable difference in secondary treatment modalities and encouraging future studies to compare these modalities in terms of complications, remission, and cost. In the end, to decide whether acromegaly patients need further therapy after transsphenoidal surgery, clinicians must be aware of the current standards for acromegaly cures. 

## Conclusion

To the best of our knowledge, this systematic review is the first report comparing remission rates between different treatment modalities in recurrent acromegaly. Medical therapy and radiotherapy had significantly higher remission rates than re-operation in these patients, respectively. Although a definite recommendation cannot be made regarding the optimal treatment in case of recurrence, our work should be the ground for future studies comparing these modalities in terms of remission, complication, and cost. 

## Data Availability

Data is presented in Table 1.
